# Rolf Neth (October 6, 1926 to March 17, 2020)

**DOI:** 10.1038/s41375-020-0829-6

**Published:** 2020-04-08

**Authors:** Rüdiger Hehlmann, Robert Gallo, Dieter Hoelzer, Karl Welte, Robert Peter Gale, Axel Zander

**Affiliations:** 1 Medical Faculty Mannheim, Heidelberg University, Mannheim, Germany; 2 ELN Foundation, Weinheim, Germany; 3 Institute of Human Virology, Baltimore, MD USA; 4 Onkologikum Frankfurt, Frankfurt, Germany; 5 Tübingen University, Tübingen, Germany; 6 Imperial College, London, UK; 7 Hamburg University, Hamburg, Germany

**Keywords:** Leukaemia, Cancer genetics


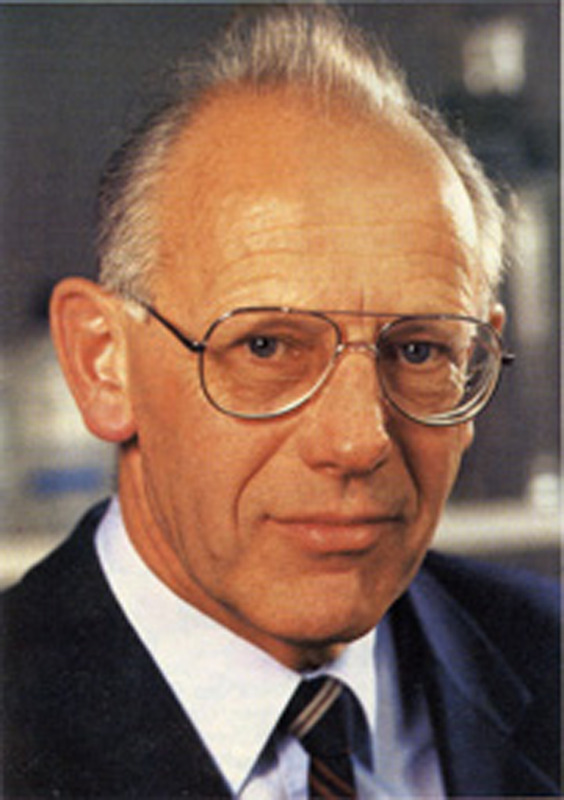


The international leukemia community lost a great influencer. Prof. Dr med. Rolf Neth died on March 17, age 93 years in his home town Buchholz near Wilsede/Lüneburger Heide. His name is intimately connected with the meetings in Wilsede on *Modern Trends in Human Leukemia* [1, 2].

Wilsede is a little village in the midst of the Lüneburger heath about an hour from Hamburg, but the Wilsede meetings which took place beginning in 1973 formed generations of leukemia researchers and beyond. The meetings were unconventional (reflecting Rolf’s unconventional and charismatic personality), and held at the end of June in an old barn, “de Emhoff”, younger investigators slept under youth hostel-like conditions, the place could not be reached by car, only by carriage, bicycle, or walking and, most importantly, could not be easily left. Sessions ended rarely on time, and informal discussions frequently lasted well beyond midnight and sometimes ended in an early morning walk through the Lüneburger heath.

The mission of the meetings was to bring young curious researchers together with internationally renowned celebrities under relaxed conditions (a setting almost impossible at the time) to discuss modern trends in leukemia research and related topics. Leukemia was selected as a specific topic because it was timely for translational research (though the term was not then used) and because samples being available would better draw various basic science areas together with clinical scientists. Rolf’s concept was unique and extremely successful.

Rolf Neth was born on October 6, 1926. In 1944, age 18 years, he was drafted to World War II and lost both forefeet. He studied medicine in Göttingen and Hamburg and got his postdoctoral training at the Max-Planck Institut für Experimentelle Medizin in Göttingen. He then trained as a pediatrician at the Children Hospital Hamburg passing his boards in 1967. He was Professor there from 1972 until his retirement in 1992. He is survived by his wife of 62 years Hannelore and four sons.

RH met Rolf first in 1972 during postdoctoral training in New York when Rolf visited Spiegelman’s laboratory at Columbia University. Rolf was touring the United States to convince leukemia researchers to support his idea of a meeting in Wilsede which he had developed with RG as a natural replacement of the Cold Spring Harbor phage meeting. RH remembers that he initially was unsure how serious the idea was, but Rolf with his insisting, convincing, and charismatic personality was able to solicit support for his concept from multiple local sources and from NIH—and succeeded.

In 1973, about 60 researchers, mostly molecular biologists or retrovirologists and some clinical trialists, came (by carriage) to the first Wilsede meeting which Rolf organized personally supported only by his three young lady assistants and—in the background—by his wife. At that time, reverse transcriptase had been detected not so long ago which reinforced the search for a human leukemia virus. Key suspect was a retrovirus. The search did not detect such virus in the common types of human leukemia, but identified growth factors, multiple oncogenes, endogenous retroviruses also in man, and ultimately the AIDS virus. Various animal leukemia viruses, immune deficiencies associated with some of these viruses and graft-versus-leukemia effects among other topics were dicussed at great length and with unrestrained enthusiasm. Wilsede provided the platform for a broad interdisciplinary discussion of this research.

Among the scientists of the first hour, RH remembers Fred Stohlman, Howard Temin, Peter Hans Hofschneider, Robert Gallo, Peter Duesberg, Arséne Burny, Harald zur Hausen, Dieter Huhn, Dieter Hoelzer, Tim Hunt, Reinhard Kurth, Roland Mertelsmann, and Ron McCaffrey.

Later many others came, among them Don Metcalf, Malcolm Moore, Leo Sachs, Donnall Thomas, Sol Spiegelman, George Todaro, Ulf Rapp, Henry Kaplan, Geoge and Eva Klein, Volker Diehl, Theodor Fliedner, Janet Rowley, Ken McCredie, Mel Greaves, Avrion Mitchison, Hans Stauss, Donald Pinkel, Edward Henderson, William Moloney, Peter Wiernik, Michael Dexter, Rudolf Jaenisch, Fritz Anders, Robin Weiss, Boris Lapin, Boris Afanasiev, Volker Erfle, Max Essex, Bill Haseltine, Karl Welte, Philip Fialkow, Robert Gale, John Goldman, and many many others. Many Nobel prizes were awarded to the field.

The second meeting took place in 1975, the third in 1978 (to alternate with the symposia of the International Association of Comparative Research on Leukemia and Related Disorders), and then continued every 2 years. Rolf organized the Wilsede meetings himself for more than 20 years and imprinted his spirit of discussion, communication, and interaction on the participants until AZ took over.

After the fall of the iron curtain Rolf extended his mission to Russia and organized the legendary Wilsede—Volga meeting on a boat cruising the Volga river. Rolf died the same week as Boris Afanasiev with whom he organized the Wilsede-Neva meetings.

As kind and helpful as Rolf usually was, he could get really angry if he thought his mission was in danger and his rules were not followed. But he calmed down quickly if he was promised improvement.

Everyone who was in Wilsede remembers Rolf’s Wilsede meetings among thousand others they may have attended before and afterwards.
