# Publisher Correction: Rolf Neth (October 6, 1926–March 17, 2020)

**DOI:** 10.1038/s41375-021-01356-5

**Published:** 2021-11-18

**Authors:** Rüdiger Hehlmann, Robert Gallo, Dieter Hoelzer, Karl Welte, Robert Peter Gale, Axel Zander

**Affiliations:** 1 Medical Faculty Mannheim, Heidelberg University, Mannheim, Germany; 2 ELN Foundation, Weinheim, Germany; 3 Institute of Human Virology, Baltimore, MD USA; 4 Onkologikum Frankfurt, Frankfurt, Germany; 5 Tübingen University, Tübingen, Germany; 6 Imperial College, London, UK; 7 Hamburg University, Hamburg, Germany

**Keywords:** Leukaemia, Cancer genetics

Correction to: *Leukemia* 10.1038/s41375-020-0829-6, published online 08 April 2020

The article Rolf Neth (October 6, 1926–March 17, 2020), written by Rüdiger Hehlmann, Robert Gallo, Dieter Hoelzer, Karl Welte, Robert Peter Gale & Axel Zander, was originally published electronically on the publisher’s internet portal on 08 April 2020 without open access. With the author(s)’ decision to opt for Open Choice the copyright of the article changed on 26. May 2021 to © The Author(s) 2021, the article is forthwith distributed under a Creative Commons Attribution

